# Child and Adolescent Suicide Rates and Economic Crisis in South Korea using Hierarchical Age-Period-Cohort Analysis

**DOI:** 10.1192/j.eurpsy.2023.1183

**Published:** 2023-07-19

**Authors:** J. Song, B. R. Roh, H. J. Hong

**Affiliations:** 1 Deparment of Psychiatry, National Health Insurance Service Ilsan Hospital, Goyang; 2 Deparment of Social Welfare, Semyung University, Jecheon; 3 Deparment of Psychiatry, Hallym University Sacred Heart Hospital, Anyang, Korea, Republic Of

## Abstract

**Introduction:**

Suicide is a complex problem in which individual, family social factors are interrelated. The 1997 Asian financial crisis caused a major economic crisis in Korea, and Korea received bailout support from the International Monetary Funds(IMF) from December 23, 1997 to August 23, 1997.

**Objectives:**

This study aimed to investigate the relationship between the suicide rate of children and adolescents who grew up during this economic crisis in Korea.

**Methods:**

Suicide rates are calculated according to gender, region, and age of 5 years (10-14 years old, 15-19 years old, 20-24 years old) using suicide death data from the Korea National Statistical Office from 2000 to 2017. The cohort of interest in the study is the group that was in childhood and early adolescence between 1997-2000 and corresponds to 1986-1995 in terms of birth year. Cohorts are divided into 1986-1989 (G1), 1990-1992 (G2), and 1993-1995 (G3) according to birth year. These groups were 8-14 years old for G1 and 5-10 years old for G2, 2-7 years old for G3 during 1997-2000, during the economic crisis. The Age-Period-Cohort analysis and linear mixed-effects regression models are used and the moderating effect on region and age is also analyzed.

**Results:**

The 10-24 year-old suicide rate was higher in males than females, in older age groups, earlier in birth years in the birth cohort, and in rural than urban areas. Suicide rates between the ages of 20-24 years were particularly high among men living in rural areas. During the national economic crisis, the suicide rate was higher among adolescents than preschoolers (G3 < G1) (p<0.001), and this trend was observed for both men and women. However, the main effect of the cohort was not observed at a statistically significant level.

**Conclusions:**

The national economic crisis has a negative impact on the mental health of children and adolescents, and it is more negative for adolescents than for children, which can increase the suicide rate between the ages of 20-24.

**Disclosure of Interest:**

None Declared

